# Associations of Computed Tomography Image-Assessed Adiposity and Skeletal Muscles with Triple-Negative Breast Cancer

**DOI:** 10.3390/cancers14071846

**Published:** 2022-04-06

**Authors:** Livingstone Aduse-Poku, Jiang Bian, Dheeraj R. Gopireddy, Mauricio Hernandez, Chandana Lall, Sara M. Falzarano, Shahla Masood, Ara Jo, Ting-Yuan David Cheng

**Affiliations:** 1 Department of Epidemiology, College of Public Health & Health Professions & College of Medicine, University of Florida, Gainesville, FL 32603, USA; adusepokul@ufl.edu; 2 Department of Health Outcomes & Biomedical Informatics, College of Medicine, University of Florida, Gainesville, FL 32610, USA; bianjiang@ufl.edu; 3 Department of Radiology, College of Medicine—Jacksonville, University of Florida, Jacksonville, FL 32209, USA; dheerajreddy.gopireddy@jax.ufl.edu (D.R.G.); mauricio.hernandez@jax.ufl.edu (M.H.); chandana.lall@jax.ufl.edu (C.L.); 4 Department of Pathology, Immunology and Laboratory Medicine, College of Medicine, University of Florida, Gainesville, FL 32610, USA; sfalzarano@ufl.edu; 5 Department of Pathology and Laboratory Medicine, College of Medicine—Jacksonville, University of Florida, Jacksonville, FL 32210, USA; shahla.masood@jax.ufl.edu; 6 Department of Health Services Research, Management & Policy, College of Public Health & Health Professions, University of Florida, Gainesville, FL 32609, USA; ara13j@phhp.ufl.edu

**Keywords:** adipose tissues, skeletal muscles, computed tomography, body composition, triple-negative breast cancer

## Abstract

**Simple Summary:**

Despite BMI’s wide use as a measure of body size and its clinical and epidemiological utility, it measures weight and height without differentiating between adipose tissue and skeletal muscle. To overcome these limitations, we analyzed the third lumbar (L3) computed tomography (CT) images of 350 breast cancer patients to measure the areas of adipose tissue and five-level skeletal muscle components and assessed their relationships with triple-negative breast cancer (TNBC), an aggressive subtype leading to higher mortality. We found that higher areas of adipose tissues were associated with an increased likelihood of TNBC subtype, especially in premenopausal women. Since the risk factors associated with adiposity and skeletal muscles are modifiable, such as healthy diets, resistance training, and adequate protein intake, it is expected that a better understanding of this body composition component will lead to novel prevention and management strategies for TNBC.

**Abstract:**

Obesity measured by anthropometrics is associated with increased risk of triple-negative breast cancer (TNBC). It is unclear to what extent specific adipose tissue components, aside from muscle, are associated with TNBC. This retrospective study included 350 breast cancer patients who received treatment between October 2011 and April 2020 with archived abdominal or pelvic computed tomography (CT) images. We measured the areas of adipose tissue and five-density levels of skeletal muscle on patients’ third lumbar vertebra (L3) image. Logistic regression was performed to examine the associations of specific adiposity and skeletal muscles components and a four-category body composition phenotype with the TNBC subtype. Results showed that higher vs. lower areas (3rd vs. 1st tertiles) of visceral adipose tissue (VAT) and subcutaneous adipose tissue (SAT) were associated with increased odds of TNBC vs. non-TNBC after adjusting for age, race, stage, tumor grade, tumor size, and skeletal muscle areas (adjusted odds ratio [AOR], 11.25 [95% CI = 3.46–36.52]) and (AOR, 10.34 [95% CI = 2.90–36.90]) respectively. Higher areas of low density muscle was also associated with increased odds of TNBC (AOR, 3.15 [95% CI = 1.05–10.98]). Compared to normal body composition (low adipose tissue/high muscle), high adiposity/high muscle was associated with higher odds of TNBC (AOR, 5.54 [95% CI = 2.12–14.7]). These associations were mainly in premenopausal women and among patients with the CT performed after breast cancer surgery. Specific adipose tissue and low-density muscle can be associated with the TNBC subtype in breast cancer patients. The direction of association warrants confirmation by prospective studies.

## 1. Introduction

Breast cancer is the most common cancer diagnosis worldwide in women [1]. In the United States, breast cancer accounts for 30% of all female cancer cases [2]. Among molecular subtypes based on protein expression status, triple-negative breast cancer (TNBC), i.e., lack of expression in estrogen receptor (ER), progesterone receptor (PR), and human epidermal growth factor receptor 2 (HER2), accounts for up to 15% of breast cancer cases [1]. TNBCs are generally more aggressive, result in poorer prognosis, and are more likely to metastasize into the liver and lungs compared to other subtypes [3].

Epidemiological studies indicate that obesity, as measured by body mass index (BMI), is positively associated with increased risk of TNBC [4,5,6]. However, the association between body size, which is in part measured by BMI, and risk of TNBC appears to differ between premenopausal and postmenopausal women. In postmenopausal women, most studies have found no association [7,8,9,10] or a reduced risk with increased BMI and body fatness [11,12]. However, in a systematic review and meta-analysis, premenopausal women with BMI ≥ 30 kg/m^2^ had a higher risk of developing TNBC compared to non-obese women [4]. Also, in premenopausal women, an increase in abdominal adiposity assessed with waist circumference, hip circumference, and waist-to-hip ratio (WHR) has been shown to increase the risk of TNBC subtype [12,13].

While BMI is commonly used as a measure of body size and has excellent clinical and epidemiological utility, limitations include the measurement of weight and height without differentiating between adipose and muscle tissue as well as other components in the body. Low BMI may mask excess adipose tissue, and high BMI may mask high skeletal muscle mass [14]. To overcome these limitations, recent studies have recommended the use of computed tomography (CT) image-assessed adiposity and skeletal muscle density as a measure of body composition [15,16,17], a technique that is now recognized as the gold standard in measuring body composition [18]. With semi-automated programming, one can accurately measure the areas of subcutaneous adipose tissue (SAT), visceral adipose tissue (VAT), intramuscular adipose tissue (IMAT), and various densities of skeletal muscles in a single CT slice in the abdominal area [19].

To our knowledge, no study has yet examined the association between CT-assessed body composition and TNBC subtype (versus non-TNBC subtype) in breast cancer patients. To address this knowledge gap, we used clinically acquired CT scans to investigate the relationship between adiposity, skeletal muscle densities, and TNBC subtype in a sample of breast cancer patients. We also hypothesized that the link between CT-assessed body composition and TNBC subtype (versus non-TNBC subtype) differed between premenopausal women and postmenopausal women.

## 2. Methods

### 2.1. Patient Population

**Figure 1:**
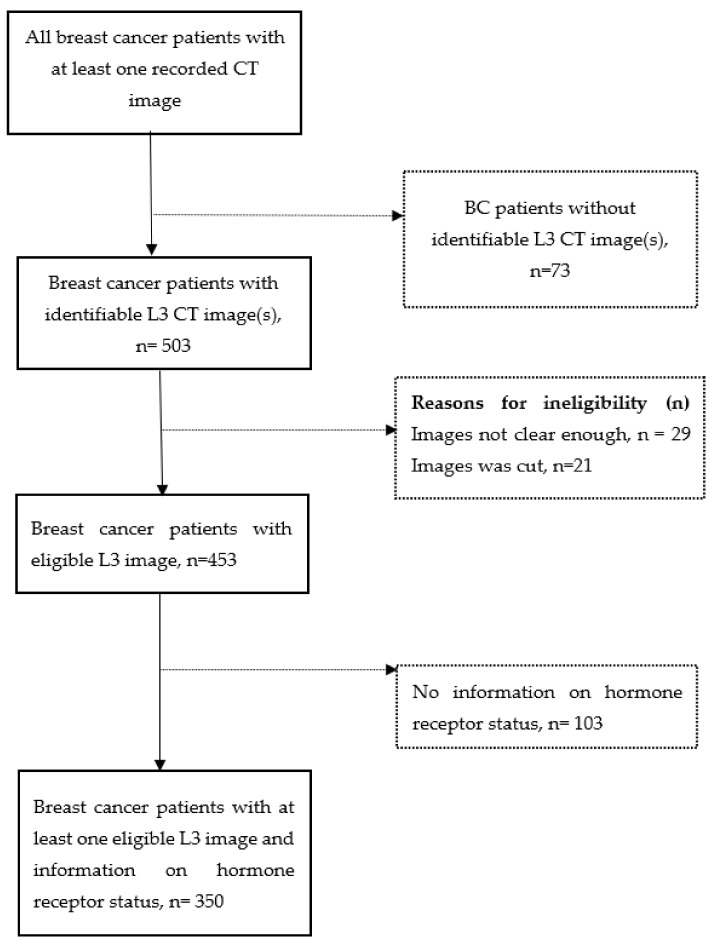
Participants selection diagram. Dash line arrow and dash line square indicate exclusion. Participants were excluded if they had missing data on either estrogen receptor status, progesterone receptor status, or human epidermal growth factor receptor 2 status.

Women diagnosed with breast cancer and received breast cancer treatment at the University of Florida Health Shands Hospital from October 2011 to April 2020, were identified through the local tumor registry linked to an electronic medical record (EMR) system. Eligible participants were women aged 20–75 years at diagnosis and with an abdominal or pelvic CT, including positron emission tomography-computed tomography (PET-CT) scan. In our sample, the majority (78.5%) of scans were taken after the breast cancer diagnosis, consistent with the fact that CT scans are prescribed for staging breast cancer. For patients with multiple CT images, the priority was to select a scan prior to and closest to breast cancer surgery. We excluded patients with a history of diabetes because diabetes and its treatment may influence obesity-related signaling pathways [20,21]. A total of 576 breast cancer patients had at least one recorded CT image. Out of these, we excluded breast cancer patients without an identifiable image at the 3rd lumbar vertebra (L3) (n = 73), without an eligible L3 CT image, i.e., unclear, incomplete, or distorted images (n = 50), and those with no information on hormone receptor status (n = 103), resulting in a final sample of 350 (Figure 1). Among the sample, 121 (35.2%) of women had body composition assessed pre-surgery, the average time from their CT scan to surgery was 15.20 ± 16.8 months. 223 (64.8%) women had body composition assessed post-surgery, and the average time from surgery to CT was 20.70 ± 20.7 months. The protocol was approved by the Institutional Review Board of the University of Florida.

### 2.2. Body Composition Measurements

A single trained investigator with the guidance of two radiologists analyzed the CT images using Sliceomatic version 5.0 revision 7 (Tomovision, Montreal, QC, Canada). Adipose tissues were identified within and around the subcutaneous region, skin, inter-muscular fat, marrow, and fat around organs [22]. We used Hounsfield unit (HU) thresholds, representing the physical properties of tissues expressed in numerical form, to further segment the different types of adipose and muscle tissues. A protocol for CT image annotation was developed using their HU ranges which were keyed into a script to aid in the automaticity of segmentation and annotation of the L3 images. Areas from single-slice abdominal images at the L3 vertebra are strongly correlated with whole-body volumes of skeletal muscles and adipose tissues and have been reported in previous studies [23]. Adipose tissues comprise SAT, IMAT, and VAT. SAT was selected by limiting the measurements to the lower attenuation of −190 HU and the upper limit of −30 HU. IMAT and VAT were limited to the HU, ranging from −190 HU to −30 HU and 150 HU to −50 HU, respectively. Total Adipose tissue (TAT) area was calculated as the summation of SAT, IMAT, and VAT areas.

The skeletal muscles component of body composition was divided based on their densities. They were classified into very-low-density muscles (VLDM), low-density muscle (LDM), normal density muscle (NDM), high-density muscle (HDM), and very high-density muscle (VHDM). VLDM, LDM, and NDM were limited with the range of −29 to 0 HU, 0 to 35 HU, and 35 to 101 HU, respectively. The range for HDM was 101–151 HU, and 151 to 200 HU was calibrated as the range for VHDM. Total skeletal muscle (TSM) area was calculated as the summation of all muscle areas of the five levels of density.

### 2.3. Triple-Negative Breast Cancer Subtype

Information on tumor characteristics such as ER status, PR status, and HER2 status was retrieved from the local tumor registry or patients’ EMR. TNBC subtype was computed from ER, PR, and HER2 statuses.

### 2.4. Covariates Assessment

Demographic information on age at diagnosis, race, weight, and height was collected from EMR. If multiple data points on weight and height were available, the measurements that were closest to breast cancer diagnosis were selected. Tumor characteristics included in the dataset were tumor stage, grade, tumor size, tumor extension, lymph node status, and recurrence. Time of breast cancer diagnosis, surgery, and CT scans were also extracted from EMR.

### 2.5. Statistical Analysis

We first examined the relationship between patient, tumor, and body composition characteristics and TNBC subtype through chi-square analysis. Patients were grouped based on the tertile of each adiposity and skeletal muscle component. In addition, we categorized patients into four mutually exclusive phenotypes based on the levels of TAT and TSM. These groups were (i) normal (high muscle/low adiposity), i.e., highest two tertiles of TSM and lowest two tertiles of TAT; (ii) high muscle/high adiposity, i.e., highest tertile of TAT and highest two tertiles of TSM (iii) low adiposity/low muscle, i.e., lowest two tertiles of TAT and lowest tertile of TSM, and (iv) high adiposity/low muscle, i.e., 3rd tertile TAT and 1st tertile TSM [24]. To examine the associations of adiposity and skeletal muscle areas (the exposure variables) with TNBC subtype (the outcome variable), we first performed a univariate logistic regression, i.e., Model 1, analysis to calculate the unadjusted odds ratio (OR) and 95% confidence intervals (CIs). We then performed a multivariable logistic regression adjusting for age (<50 y, ≥50 y), race (White, Black American), tumor stage (0, I, II, III, IV), and tumor size (<2 cm, ≥2 cm), i.e., Model 2. In a separate model, in addition to the previously adjusted covariates, we adjusted for TAT (in tertiles) when modeling skeletal muscles and for TSM (in tertiles) when modeling adiposity, i.e., Model 3. Also, we stratified the study participants into premenopausal (age < 55 years) and postmenopausal (age ≥ 55 years) women to examine the association between body composition and TNBC subtype. A stratified analysis was also performed on CT scans taken before and after breast cancer surgery because body composition based on a CT scan taken before surgery was more consistent with the temporality compared to that taken after surgery. All statistical analyses were performed using SAS software, Version 9.4 (SAS Institute Inc., Cary, NC, USA, 2017) [25].

## 3. Results

Our study participants consisted of 350 breast cancer patients who had data on hormone receptor status; 24.6% of these participants received a diagnosis of TNBC. Overall, the mean age of the participants was 58.5 ± 9.6. The prevalence of obesity and overweight were 34.7% and 33.3% respectively. Patients with non-TNBC subtypes had higher proportion of obesity (BMI > 30 kg/m^2^) than those with TNBC subtype (34.7% vs. 18.4%; *p* = 0.027; Table 1). A majority of the study participants were White (86.0%); the proportion of Blacks in patients with the TNBC subtype (19.8%) was higher than those without TNBC (12.1%). Patients with TNBC vs. those with non-TNBC had a higher percentage of stage III/IV diseases (30.1% vs. 15.1%), grade III tumors (85.9% vs. 42.8%), and larger tumors (84.0% vs. 32.0%) (all *p* < 0.05).

Regarding body composition, the distributions of visceral adipose tissue, total adipose tissue, and total skeletal muscle in tertiles were significantly different in participants with TNBC subtype compared to those without TNBC subtype *(p* < 0.05). Body composition categories were significantly different between participants with and without TNBC subtype (*p* = 0.037); the proportion of individuals with the high adiposity/low muscle phenotype was 4.9% in TNBC patients and 7.1% in non-TNBC patients (Table 1).

Table 2 shows the regression analysis results for the associations between specific body composition components and the TNBC subtype. In the unadjusted model, high (T3) vs. low (T1) SAT was not associated with TNBC subtype (OR = 1.28 [95% CI = 0.71–2.31], Model 1). In the adjusted model, high vs. low of SAT was associated with higher odds of TNBC subtype after adjusting for age, race, tumor stage, tumor grade, and tumor size (adjusted odds ratio [AOR], 6.10 [95% CI = 1.93–19.32], Model 2). The association was stronger after additionally adjusting for TSM areas (AOR, 10.34 [95% CI = 2.90–36.90], Model 3). The associations were similar for VAT (AOR, 11.25 [95% CI = 3.46–36.25], Model 3) and TAT (AOR = 3.99 [95% CI = 1.37–11.68], Model 3). Also, middle vs. low VAT was associated with higher odds of TNBC subtype after adjusting for age, race, tumor stage, tumor grade, and tumor size (adjusted odds ratio [AOR], 6.72 [95% CI = 2.12–21.36], Model 2); this association was stronger after further adjusting for TSMFor skeletal muscle with different densities, high vs. low LDM (AOR = 4.25 [95% CI = 1.05–10.38], Model 3) was associated with increased odds of TNBC subtype. Also, middle vs. low LDM was associated with higher odds of TNBC subtype (AOR = 3.35 [95% CI = 1.11–10.10], Model 3). However, high vs. low TSM was not associated with TNBC subtype (AOR = 0.75 [95% CI = 0.29–1.96], Model 3).

For Table 3 and Table 4, participants were stratified into premenopausal and postmenopausal women, and the association between their body composition and TNBC subtype was assessed. In premenopausal women, the unadjusted model showed that high vs. low SAT (OR = 3.30 [95% CI = 1.25–8.74], Model 1), IMAT (OR = 3.82 [95% CI = 1.17–12.44], Model 1), VAT (OR = 4.16 [95% CI = 1.46–11.88], Model 1), and TAT (OR = 4.44 [95% CI = 1.53–12.90], Model 1) were associated with higher odds of TNBC subtype (Table 3). In the adjusted model, middle vs. low VAT (OR = 213 [95% CI = 14.26–603.78], model 2) and TAT (OR = 21.11 [95% CI = 2.56–174.08], model 2) were associated with increased odds of TNBC subtype. Similarly, high vs. low SAT (OR = 32.90 [95% CI= 7.27–148.98], model 2), VAT (OR = 59.91 [95% CI = 6.85–524.19], model 2), and TAT (OR = 108.64 [95% CI = 10.40–442.75], model 2) were associated with higher odds of TNBC subtype. The association was stronger after adjusting for age, race, tumor stage, tumor grade, tumor size, and TSM. Among different densities of muscle tissue, high vs. low VLDM (OR = 3.10 [95% CI = 1.14–8.47], model 1) and middle vs. low TSM (OR = 4.02 [1.27–12.71], model 1) were associated with higher odds of TNBC subtype. Also, high vs. low VLDM (AOR = 7.62 [95% CI = 1.40–41.49], Model 2), middle vs. low NDM (OR = 6.15 [95% CI = 1.16–32.75], model 2), and middle vs. low TSM (OR = 6.15 [95% CI = 1.13–33.34], model 2) were associated with higher odds of TNBC after adjusting for covariates, but there was no association after further adjusting for TAT. In postmenopausal women, adipose tissues and skeletal muscles were not associated with the TNBC subtype (Table 4).

Compared to normal body composition (low adipose tissue/high muscle), high adiposity/high muscle was associated with higher odds of TNBC subtype (OR = 2.71 [95% CI = 1.33–5.51], Model 1] (Table 5); this association was more pronounced after adjusting for age, race, tumor stage, tumor grade, tumor size (AOR = 5.54 [95% CI = 2.12–14.7], Model 2). A similar observation was made in premenopausal women. In premenopausal women, high adiposity/high muscle was associated with higher odds of TNBC subtype, compared to normal body composition (AOR = 20.92 [95% CI = 4.46–166.65], Model 2). In postmenopausal women, adipose tissues and skeletal muscles were not associated with the TNBC subtype.

We stratified the analyses by the time of CT in relation to surgery, i.e., CT scans taken before or after surgery. For patients with CT scans taken before surgery, specific body composition components were not associated with the TNBC subtype (Table S1). For those with scans taken after surgery, high vs. low SAT was associated with higher odds of TNBC subtype, (OR = 3.13 [95% CI = 1.37–7.14], Model 1) (Table S2). Also, high vs. low IMAT and VAT were associated with increased odds of TNBC subtype (OR = 3.31 [95% CI = 1.42–7.72], Model 1) and (OR = 5.59 [95% CI = 2.39–13.10], respectively. In addition, middle vs. low VAT was associated with increased of TNBC subtype (OR = 3.75 [1.68–8.37], model 1). These associations were more pronounced after adjusting for age, race, tumor stage, tumor grade, tumor size, and TSM. For skeletal muscle tissues, VLDM (OR = 7.58 [95% CI = 1.54–37.25]) was positively associated with TNBC subtype, but NDM (AOR = 0.16 [95% CI = 0.04–0.74], model 3) and TSM (AOR = 0.24 [95% CI = 0.06–0.98], Model 3) were inversely associated with the TNBC subtype, after adjusting for age, race, tumor stage, tumor grade, tumor size, and TSM. In addition, middle vs. low LDM was associated with increased odds of TNBC subtype (AOR = 7.46 [1.83–30.34], model 2). However, the 4-body composition phenotypes were not associated with TNBC after stratifying by the time of CT in relation to surgery (Table S3).

## 4. Discussion

To our knowledge, this is the first study to examine the associations of CT-assessed adiposity and skeletal muscles with TNBC subtype in breast cancer patients. Higher vs. lower areas (3rd vs. 1st tertiles) of VAT, SAT, and TAT were associated with increased odds of TNBC after adjusting for age, race, tumor stage, tumor grade, and tumor size. Compared to normal body composition (low adiposity/high muscle), high adiposity was associated with a higher odds of TNBC subtype. The associations were mainly in premenopausal women but not in postmenopausal women.

Optimal measurement of adipose tissue would require both the amount and sites of disposition of the adipose tissue [26]. Our study quantified SAT, VAT, and IMAT separately, as VAT has been seen to be more metabolically active than SAT due to its high lipolytic activity and release of large amounts of free fatty acids [27,28]. However, most studies examining the association between body size and TNBC subtype have used BMI and waist circumference measurements, which do not differentiate between adipose tissues and skeletal muscles components. CT is a highly accurate method for measuring fat distribution and skeletal muscle mass due to its ability to provide cross-sectional images which can be compartmentalized [23]. In this study, high vs. low SAT and VAT were found to be associated with the TNBC subtype. Although no previous study has been done on CT-assessed adiposity-TNBC association, research reported that higher CT-assessed abdominal distribution of VAT was associated with elevated odds of ER-negative, PR-negative, and HER2 negative breast cancers compared to ER, PR, and HER2 positive breast cancers [29]. In a population-based case-control study, there was an increased risk of developing TNBC in women with higher WHR [30]. In a case-only analysis, women with TNBC were more likely to be obese compared to those with non-TNBC subtypes [31].

Mechanisms directly linking SAT and VAT to TNBC subtype are unclear. However, studies identifying the potential mechanistic links between obesity and TNBC subtypes have suggested some mechanisms. First, obesity-mediated inflammatory cytokines, such as leptin, and activation pathways that stimulate invasion and metastasis have been suggested [32]. Circulating levels of insulin and leptin are high in obesity [33]. Insulin stimulates the overexpression of leptin which sets up an autocrine loop to stimulate breast cancer cell growth [32]. Also, the activation of mammalian target of rapamycin (mTOR) by insulin has been seen as a predictor of poor prognosis in women with TNBC [34,35,36]. Moreover, mTOR promotes a switch from mitochondrial respiration to aerobic respiration, i.e., the Warburg effect [37,38]. The Warburg effect increases glucose uptake that supplies anabolic precursors for the rapid growth of TNBCs through mitochondrial dysfunction [33]. Also, ethyl-CpG-binding domain protein 2 (MBD2) may play a role in the tumor initiation capacity in inoculated TNBC cell lines [39].

We also assessed the association between 4 mutually exclusive body composition phenotypes. We found that compared to normal body composition (low adiposity/high muscle), high adiposity was associated with higher odds of TNBC subtype. The distribution of the four body composition phenotypes was similar between our study and the previous studies of colorectal cancer patients and healthy individuals [24,40]. With the same phenotype classification, colorectal cancer patients with the high adiposity/low muscle phenotype had a 64% higher risk of overall mortality compared to those with adequate muscle and lower adiposity phenotype, signifying the importance of body composition phenotypes in assessing cancer survival [24]. However, there is no report on the association between these four phenotypes and survival in breast cancer.

In our study, the associations between adiposity and TNBC subtype were mainly seen in premenopausal women but not postmenopausal women. In premenopausal women, high vs. low SAT, IMAT, and VAT were associated with higher odds of TNBC subtype. This is in line with the findings of two large case-control studies [4,41] and a consortium of African American/Black women [12] that larger vs. smaller body size measured with WHR was associated with increased risk of TNBC among premenopausal women. We, however, found no association between adiposity and TNBC subtype in postmenopausal women. Menopausal status may be a factor mitigating the effect of body size on the incidence of TNBC [4]. Postmenopausal women often present with less aggressive phenotypes and estrogen-dependent lesions, most likely resulting from the production of steroidal hormones from adipocytes [42]. On the contrary, premenopausal women often present with more aggressive phenotypes (large size, high tumor grade, and high proliferation rate) and hormone-independent lesions [42,43].

In our sample of breast cancer patients, TSM area was not associated with TNBC subtype; however, high vs. low LDM was associated with TNBC subtype. Also in premenopausal women, high vs. low HDM was associated decreased odds of TNBC subtype. In spite of the lack of studies investigating the association between skeletal muscle areas and TNBC subtype, 6 previous studies [14,44,45,46,47,48] have assessed the association between skeletal muscle areas and survival, 3 in metastatic disease [45,46,47] and 3 in non-metastatic disease [14,44,48]. Only one study did not show an increased risk of death among breast cancer patients with low muscle mass [47]. In our study, when assessing the association between adipose tissues and TNBC subtype, further adjusting for TSM in a multivariable model increased the odds ratio of TNBC. These findings indicate that skeletal muscle areas may be an important confounder of the adiposity-TNBC relationship and should be considered in study design and analyses.

Limitations of this study include its limited generalizability beyond breast cancer patients who did not have a CT scan, the use of cross-sectional design, and relatively small sample size in the stratified analyses. CT images were obtained based on availability, so a high proportion of CT scans were taken after breast cancer surgery. When we stratified the analysis by the time of CT scan taken, i.e., pre-or post-surgery, we found that the associations were mainly in scans taken after the surgery. The reason for the discrepancy is not clear. One possibility for this observation may be due to the relatively small sample size in the group that had scans before surgery. In our post-hoc analysis of sample size, for the four-categories body composition, comparing high adiposity/low muscle group to normal group, one would need 34 TNBC cases and 104 controls to have sufficient power for the effect overall (effect size = 4.87, alpha = 0.05, power = 80%) and 31 cases and 128 controls for the group that had scans before surgery for the overall effect of (effect size = 3.13, alpha = 0.05, power = 80%). It is also unclear if the pre-surgery scans were taken in the window that can influence breast cancer subtypes. After surgery, cancer patients are usually less mobile and less physically active, which may lead to fat accumulation and skeletal muscle loss. Also, our data lacked information on chemotherapy as well as anti-lipid therapy, both of which may influence body composition. Patients under statin therapy seem to be more vulnerable to the aging-associated lowering of fat-free mass in a prospective cohort study [49]. In a randomized controlled trial, statins decreased HUs of epicardial adipose tissues over one year, although there was no change in subcutaneous adipose tissue HUs [50]. Despite these limitations, we used L3 images from CT scans which provide more details on the extent and location of adipose tissues and muscle mass, all of which provide different profiles of metabolic markers that may be associated with the TNBC subtype.

## 5. Conclusions

We demonstrate that adiposity measured by L3 CT images is associated with the TNBC subtype, especially in premenopausal breast cancer patients. This finding supports the need for prospective studies with larger sample sizes and non-breast cancer controls evaluating body composition and risk of TNBC. As adiposity and muscle mass are modifiable risk factors in cancer patients, e.g., diet-induced weight-loss interventions, resistance training, and adequate protein intake, a better understanding of these body composition components may lead to novel preventive strategies and contribute to the management of TNBC.

## Figures and Tables

**Table 1:** Patient and tumor characteristics by Triple-Negative Breast Cancer (TNBC) subtype (N = 350).

Variables	Total (N = 350)	TNBC (N = 86, 24.6%)	Non-TNBC (N = 264, 75.4%)	*p*-Value
**Age**				
<55 years	126 (37.8)	37 (44.6)	89 (35.6)	0.183
≥55 years	207 (62.2)	46 (54.4)	161 (64.4)	
Missing	17	3	14	
Missing				
**Body Mass Index**				
Underweight (<18.5)	1 (0.3)	0 (0.00)	1 (0.4)	0.027 *
Normal weight (18.5–24.9)	95 (31.7)	34 (45.3)	61 (27.2)	
Overweight (25.0–29.9)	100 (33.3)	22 (29.3)	78 (34.7)	
Obese (30 and above)	104 (34.7)	19 (18.4)	85 (34.7)	
Missing	50	11	39	
Missing				
**Race**				
White	301 (86.0)	69 (80.2)	232 (87.9)	0.076
Black American	49 (14.0)	17 (19.8)	32 (12.1)	
**Disease stage**				
0	3 (0.9)	3 (3.6)	0 (0.0)	<0.001 *
I	144 (43.0)	24 (28.9)	120 (47.6)	
II	125 (37.3)	31 (37.4)	94 (37.3)	
III	45 (13.4)	23 (27.7)	22 (8.7)	
IV	18 (5.4)	2 (2.4)	16 (6.4)	
Missing	15	3	12	
**Tumor grade**				
I	38 (11.4)	1 (1.2)	37 (14.8)	<0.001 *
II	117 (34.9)	11 (12.9)	106 (42.4)	
III	180 (53.7)	73 (85.9)	107 (42.8)	
Missing	15	1	14	
**Tumor size**				
<2 cm	168 (54.4)	13 (16.0)	155 (68.0)	<0.001 *
≥2 cm	141 (45.6)	68 (84.0)	73 (32.0)	
Missing	41	5	36	
**Subcutaneous Adipose Tissue**				
Low (tertile 1)	99 (32.4)	23 (28.4)	76 (31.7)	0.39
Middle (tertile 2)	104 (33.0)	28 (34.6)	76 (31.7)	
High (tertile 3)	118 (34.6)	30 (37.0)	88 (36.6)	
Missing	29	5	24	
**Intramuscular Adipose Tissue**				
Low (tertile 1)	105 (32.7)	31 (38.3)	74 (30.8)	0.434
Middle (tertile 2)	107 (33.3)	26 (32.1)	81 (33.8)	
High (tertile 3)	109 (34.0)	24 (29.6)	85 (35.4)	
Missing	29	5	24	
**Visceral Adipose Tissues**				
Low (tertile 1)	94 (30.8)	22 (27.2)	72 (30.0)	0.021 *
Middle (tertile 2)	99 (34.3)	21 (25.9)	78 (32.5)	
High (tertile 3)	128 (34.9)	38 (46.9)	90 (37.5)	
Missing	29	5	24	
**Very Low-Density Muscle**				
Low (tertile 1)	99 (30.9)	29 (35.8)	70 (29.2)	0.378
Middle (tertile 2)	108 (33.6)	28 (34.6)	80 (33.3)	
High (tertile 3)	114 (35.5)	24 (29.6)	90 (37.5)	
Missing	29	5	24	
**Low Density Muscle**				
Low (tertile 1)	98 (30.5)	29 (35.80)	69 (28.75)	0.352
Middle (tertile 2)	110 (34.3)	23 (28.40)	87 (36.25)	
High (tertile 3)	113 (35.2)	29 (35.80)	84 (35.00)	
Missing	29	5	24	
**Normal Density Muscle**				
Low (tertile 1)	112 (34.9)	23 (28.4)	89 (37.1)	0.29
Middle (tertile 2)	105 (32.7)	27 (33.3)	78 (32.5)	
High (tertile 3)	104 (32.4)	31 (38.3)	73 (30.4)	
Missing	29	5	24	
**High Density Muscle**				
Low (tertile 1)	98 (30.5)	25 (30.9)	73 (30.4)	0.719
Middle (tertile 2)	102 (31.8)	23 (28.4)	79 (32.9)	
High (tertile 3)	121 (37.7)	33 (40.7)	88 (36.7)	
Missing	29	5	24	
**Very High-Density Muscle**				
Low (tertile 1)	106 (33.0)	28 (34.6)	78 (32.5)	0.587
Middle (tertile 2)	97 (30.2)	27 (33.3)	70 (29.2)	
High (tertile 3)	118 (36.8)	26 (32.1)	92 (38.3)	
Missing	29	5	24	
**Total Adipose Tissue**				
Low (tertile 1)	93 (32.7)	16 (19.75)	77 (32.08)	0.016 *
Middle (tertile 2)	107 (33.3)	33 (40.74)	74 (30.83)	
High (tertile 3)	121 (34.0)	32 (39.51)	89 (37.08)	
Missing	29	5	24	
**Total Skeletal Muscle**				
Low (tertile 1)	103 (32.2)	27 (33.3)	76 (31.8)	0.005 *
Middle (tertile 2)	106 (33.1)	16 (19.8)	90 (37.7)	
High (tertile 3)	112 (34.7)	38 (46.9)	74 (30.5)	
Missing	29	5	24	
**Body composition categories**				
Normal (Low adiposity, high muscle)	134 (41.9)	42 (51.9)	92 (38.5)	0.037 *
High adiposity, high muscle	84 (25.9)	12 (14.8)	72 (29.7)	
Low adiposity, low muscle,	82 (25.6)	23 (28.4)	59 (24.7)	
High adiposity, low muscle	21 (6.6)	4 (4.9)	17 (7.1)	
Missing	29	5	24	

TAT area = areas of SAT + IMAT + VAT. TSM area = areas of VLDM + LDM + NDM + HDM + VHDM. TNBC: triple-negative breast cancer. * *p* < 0.05.

**Table 2:** Odds ratio (95% CI) for the association between specific body composition components and TNBC subtype.

Body Composition	Model 1	Model 2	Model 3
	Unadjusted OR (95% CI)	Adjusted OR ^a^ (95% CI)	Adjusted OR ^b^ (95% CI)
**Subcutaneous Adipose Tissue**			
Low (tertile 1)	ref	ref	ref
Middle (tertile 2)	1.01(0.55–1.87)	2.14 (0.81–5.66)	2.99 (0.99–8.97) *
High (tertile 3)	1.28 (0.71–2.31)	6.10 (1.93–19.32) *	10.34 (2.90–36.90) *
**Intramuscular Adipose Tissue**			
Low (tertile 1)	ref	ref	ref
Middle (tertile 2)	1.31 (0.71–2.40)	2.47 (0.86–7.03)	2.14 (0.72–6.35)
High (tertile 3)	1.48 (0.80–2.75)	1.57 (0.99–4.72)	1.36 (0.44–4.25)
**Visceral Adipose Tissue**			
Low (tertile 1)	ref	ref	ref
Middle (tertile 2)	1.96 (1.05–3.65)	6.72 (2.12–21.36) *	6.15 (1.89–20.00) *
High (tertile 3)	2.16 (1.17–3.97) *	6.93 (2.45–19.57) *	11.25 (3.46–36.52) *
**Very Low-Density Muscle**			
Low (tertile 1)	ref	ref	ref
Middle (tertile 2)	1.18 (0.64–2.18)	1.41 (0.52–3.86)	0.70 (0.23–2.16)
High (tertile 3)	1.55 (0.83–2.90)	1.65 (0.57–4.77)	0.33 (0.08–1.34)
**Low Density Muscle**			
Low (tertile 1)	ref	ref	ref
Middle (tertile 2)	1.59 (0.85–2.99)	3.81 (1.42–10.00) *	3.35 (1.11–10.10) *
High (tertile 3)	1.22 (0.67–2.23)	4.25 (1.56–11.59) *	3.15 (1.05- 10.38) *
**Normal Density Muscle**			
Low (tertile 1)	ref	ref	ref
Middle (tertile 2)	0.75 (0.40–1.41)	1.42 (0.50–4.02)	1.74 (0.54–5.62)
High (tertile 3)	0.61 (0.33–1.13)	0.51 (0.19–1.40)	0.69 (0.23–2.07)
**High Density Muscle**			
Low (tertile 1)	ref	ref	ref
Middle (tertile 2)	1.18 (0.61–2.25)	0.67 (0.22–1.99)	0.93 (0.28–3.13)
High (tertile 3)	0.91 (0.50–1.67)	0.39 (0.14–1.08)	0.48 (0.15–1.50)
**Very High-Density Muscle**			
Low (tertile 1)	ref	ref	ref
Middle (tertile 2)	0.93 (0.50–1.73)	0.61 (0.23–1.63)	0.62 (0.21–1.83)
High (tertile 3)	1.27 (0.69–2.35)	0.99 (0.38–2.60)	0.56 (0.18–1.71)
**Total Adipose Tissue**			
Low (tertile 1)	ref	ref	ref
Middle (tertile 2)	0.93 (0.52–1.67)	1.72 (0.72–4.14)	1.71 (0.68–4.27)
High (tertile 3)	2.31 (1.18–4.53) *	3.53 (1.28–9.72) *	3.99 (1.37–11.68) *
**Total Skeletal Muscle**			
Low (tertile 1)	ref	ref	ref
Middle (tertile 2)	2.00 (1.00–3.98)	1.44 (0.54–3.86)	1.26 (0.45–3.55)
High (tertile 3)	0.68 (0.38–1.23)	1.13 (0.46–2.77)	0.75 (0.29–1.96)

^a^ Model 2 is adjusted for age, race, disease stage, tumor grade, and tumor size. ^b^ Model 3 is adjusted for age, race, disease stage, tumor grade, tumor size, and adipose tissue (for skeletal muscles) or muscle (for adipose tissue). TAT area = areas of SAT + IMAT + VAT. TSM area = areas of VLDM + LDM + NDM + HDM + VHDM. TNBC: triple-negative breast cancer. Ref indicates reference group. * *p* < 0.05.

**Table 3:** Odds ratio (95% CI) for the association between specific body composition components and TNBC subtype among premenopausal women (<55 years).

Body Composition Variables	Model 1	Model 2	Model 3
	Unadjusted OR (95% CI)	Adjusted OR ^a^ (95% CI)	Adjusted OR ^b^ (95% CI)
**Subcutaneous Adipose Tissue**			
Low (tertile 1)	ref	ref	ref
Middle (tertile 2)	1.66 (0.63–4.37)	2.05 (0.69–6.12)	3.25 (0.50–20.98)
High (tertile 3)	3.30 (1.25–8.74) *	32.90 (7.27–148.98) *	34.07 (4.58–253.31) *
**Intra-Muscular Adipose Tissue**			
Low (tertile 1)	ref	ref	ref
Middle (tertile 2)	1.41 (0.56–3.50)	4.41 (0.95–20.45)	4.13 (0.80–21.21)
High (tertile 3)	3.82 (1.17–12.44) *	4.06 (0.72–22.85)	3.75 (0.61–23.01)
**Visceral Adipose Tissue**			
Low (tertile 1)	ref	ref	ref
Middle (tertile 2)	5.28 (1.88–14.86) *	213.75 (14.26–603.78) *	160.24 (10.29–440.32) *
High (tertile 3)	4.16 (1.46–11.88) *	59.91 (6.85–524.19) *	106.71 (8.61–356.43) *
**Very Low-Density Muscle**			
Low (tertile 1)	ref	ref	ref
Middle (tertile 2)	1.63 (0.64–4.17)	5.00 (1.04–24.12) *	0.97 (0.15–6.44)
High (tertile 3)	3.10 (1.14–8.47) *	7.62 (1.40–41.49) *	0.62 (0.06–6.73)
**Low-Density Muscle**			
Low (tertile 1)	ref	ref	ref
Middle (tertile 2)	2.20 (0.83–5.83)	4.20 (0.98–17.98)	4.26 (0.44–41.77)
High (tertile 3)	1.63 (0.64–4.16)	3.61 (0.84–15.56)	0.46 (0.04–4.81)
**Normal Density Muscle**			
Low (tertile 1)	ref	ref	ref
Middle (tertile 2)	0.82 (0.26–2.54)	6.15 (1.16–32.75) *	13.87 (0.83–230.91)
High (tertile 3)	0.42 (0.14–1.22)	0.41 (0.08–2.14)	0.25 (0.02–3.32)
**High Density Muscle**			
Low (tertile 1)	ref	ref	ref
Middle (tertile 2)	1.43 (0.52–3.93)	0.43 (0.08–2.27) *	1.04 (0.13–8.28)
High (tertile 3)	0.58 (0.23–1.45)	0.14 (0.03–0.67) *	0.33 (0.05–2.09)
**Very-High Density Muscle**			
Low (tertile 1)	ref	ref	ref
Middle (tertile 2)	0.80 (0.30–2.13)	0.51 (0.12–2.23)	0.33 (0.05–2.39)
High (tertile 3)	0.97 (0.39–2.41)	0.46 (0.13–1.70)	0.50 (0.08–3.22)
**Total Adipose Tissue**			
Low (tertile 1)	ref	ref	ref
Middle (tertile 2)	1.42 (0.57–3.56)	21.11 (2.56–174.08) *	15.64 (1.52–161.18) *
High (tertile 3)	4.44 (1.53–12.90) *	108.64 (10.40–442.75) *	194.58 (13.45–367.34) *
**Total Skeletal Muscle**			
Low (tertile 1)	ref	ref	ref
Middle (tertile 2)	4.02 (1.27–12.71) *	6.15 (1.13–33.34) *	5.91 (0.53–65.67)
High (tertile 3)	1.00 (0.41–2.46)	1.04 (0.24–4.42)	0.32 (0.04–2.85)

^a^ Model 2 is adjusted for age, race, disease stage, tumor grade, and tumor size. ^b^ Model 3 is adjusted for age, race, disease stage, tumor grade, tumor size, and TAT (for skeletal muscles) or TSM (for adipose tissue). TAT area = areas of SAT + IMAT + VAT. TSM area = areas of VLDM + LDM + NDM + HDM + VHDM. TNBC: triple-negative breast cancer. Ref indicates reference group. * *p* < 0.05.

**Table 4:** Odds ratio (95% CI) for the association between specific body composition components and TNBC subtype among postmenopausal women (≥55 years).

Body Composition Variables	Model 1	Model 2	Model 3
	Unadjusted OR (95% CI)	Adjusted OR ^a^ (95% CI)	Adjusted OR ^b^ (95% CI)
**Subcutaneous Adipose Tissue**			
Low (tertile 1)	ref	ref	ref
Middle (tertile 2)	0.79 (0.36–1.77)	1.22 (0.29–5.14)	3.11 (0.52–18.60)
High (tertile 3)	0.98 (0.42–2.28)	1.27 (0.25–6.61)	3.78 (0.54–26.59)
**Intra-Muscular Adipose Tissue**			
Low (tertile 1)	ref	ref	ref
Middle (tertile 2)	0.94 (0.37–2.37)	1.70 (0.26–10.98)	1.22 (0.17–8.89)
High (tertile 3)	0.73 (0.30–1.77)	0.58 (0.09–3.70)	0.32 (0.04–2.43)
**Visceral Adipose Tissue**			
Low (tertile 1)	ref	ref	ref
Middle (tertile 2)	0.98 (0.42–2.28)	1.00 (0.22–4.50)	1.34 (0.28–6.33)
High (tertile 3)	1.22 (0.54–2.74)	2.06 (0.52–8.16)	4.21 (0.83–21.33)
**Very Low-Density Muscle**			
Low (tertile 1)	ref	ref	ref
Middle (tertile 2)	0.76 (0.32–1.81)	0.33 (0.04–2.60)	0.27 (0.03–2.40)
High (tertile 3)	0.88 (0.37–2.14)	0.19 (0.02–1.44)	0.17 (0.02–1.50)
**Low-Density Muscle**			
Low (tertile 1)	ref	ref	ref
Middle (tertile 2)	1.21 (0.52–2.84)	1.18 (0.29–4.88)	1.70 (0.34–8.54)
High (tertile 3)	1.00 (0.44–2.27)	2.12 (0.44–10.22)	3.34 (0.59–18.82)
**Normal Density Muscle**			
Low (tertile 1)	ref	ref	ref
Middle (tertile 2)	0.67 (0.31–1.48)	0.42 (0.10–1.73)	0.68 (0.13–3.47)
High (tertile 3)	0.86 (0.37–1.98)	0.57 (0.13–2.53)	1.18 (0.15–9.19)
**High Density Muscle**			
Low (tertile 1)	ref	ref	ref
Middle (tertile 2)	0.94 (0.39–2.28)	0.72 (0.13–3.83)	0.68 (0.13–3.74)
High (tertile 3)	1.03 (0.44–2.42)	0.93 (0.20–4.29)	1.35 (0.28–6.63)
**Very-High Density Muscle**			
Low (tertile 1)	ref	ref	ref
Middle (tertile 2)	0.91 (0.40–2.09)	0.89 (0.21–3.68)	0.69 (0.15–3.17)
High (tertile 3)	1.39 (0.60–3.26)	0.58 (0.50–12.63)	1.25 (0.21–7.47)
**Total Adipose Tissue**			
Low (tertile 1)	ref	ref	ref
Middle (tertile 2)	2.23 (0.33–1.59)	2.13 (0.39–11.58)	0.47 (0.12–1.91)
High (tertile 3)	1.50 (0.62–3.64)	0.42 (0.09–2.06)	8.17 (0.89–72.29)
**Total Skeletal Muscle**			
Low (tertile 1)	ref	ref	ref
Middle (tertile 2)	1.20 (0.49–2.94)	0.69 (0.15–3.17)	0.46 (0.39–11.58)
High (tertile 3)	0.50 (0.22–1.12)	1.25 (0.21–7.47)	0.42 (0.09–2.06)

^a^ Model 2 is adjusted for age, race, disease stage, tumor grade, and tumor size. ^b^ Model 3 is adjusted for age, race, disease stage, tumor grade, tumor size, and adipose tissue (for skeletal muscles) or muscle (for adipose tissue). TAT area = areas of SAT + IMAT + VAT. TSM area = areas of VLDM + LDM + NDM + HDM + VHDM. TNBC: triple-negative breast cancer.

**Table 5:** Four-category of body composition phenotype in relation to TNBC subtype.

Body Composition Categories	Model 1 Unadjusted OR (95% CI)	Model 2
		Adjusted OR ^a^ (95% CI)
**All women**		
Normal (Low adiposity, high muscle)	ref	ref
High adiposity, high muscle	2.71 (1.33–5.51) *	5.54 (2.12–14.7) *
Low adiposity, low muscle	1.17 (0.64–2.14)	1.02 (0.37–2.83)
High adiposity, low muscle	1.94 (0.62–6.12)	4.87 (0.78–30.24)
**Premenopausal women only**		
Normal (Low adiposity, high muscle)	ref	ref
High adiposity, high muscle	4.08 (1.24–13.46) *	20.32 (4.46–166.65) *
Low adiposity, low muscle	0.75 (0.29–1.93)	0.92 (0.15–5.39)
High adiposity, low muscle	1.97 (0.37–10.50)	6.95 (0.51–94.34)
**Postmenopausal women only**		
Normal (Low adiposity, high muscle)	ref	ref
High adiposity, high muscle	2.10 (0.86–5.12)	1.89 (0.51–7.07)
Low adiposity, low muscle,	1.68 (0.73–3.88)	1.63 (0.40–6.61)
High adiposity, low muscle	1.00 (0.41–9.81)	6.21 (0.44–88.29)

^a^ Model 2 is adjusted for age, race, disease stage, tumor grade, and tumor size. * *p* < 0.05.

## Data Availability

Data will not be available to the public to protect patient privacy.

## Supplementary Materials

The following supporting information can be downloaded at: https://www.mdpi.com/article/10.3390/cancers14071846/s1, Table S1: Associations of computed tomography image-assessed adiposity and skeletal muscles with triple-negative breast cancer; Table S2: Associations of computed tomography image-assessed adiposity and skeletal muscles with triple-negative breast cancer; Table S3: Associations of computed tomography image-assessed adiposity and skeletal muscles with triple-negative breast cancer.
