# Supplementary material for: Associations of Computed Tomography Image-Assessed Adiposity and Skeletal Muscles with Triple-Negative Breast Cancer
Source: Cancers (Basel). 2022 Apr 6;14(7):1846. doi: 10.3390/cancers14071846 (PMC8997963; doi:10.3390/cancers14071846)
Supplement: Supplementary file 1 [file cancers-14-01846-s001.zip › cancers-1633019 supplementary.pdf]

Table S1. Odds ratio (OR) and 95% confidence interval (CI) for the association between specific body composition components and triple-negative breast cancer (TNBC) subtype among patients with pre-surgery computed tomography (CT) scans (N=121).

Body composition	Model 1<br>Unadjusted OR (95% CI)	Model 2<br>Adjusted OR[a] (95% CI)	Model 3<br>Adjusted OR[b] (95% CI)
**Subcutaneous Adipose Tissue**<br>Low (tertile 1<br>Middle (tertile 2)<br>High (tertile 3)	 ref<br>2.11 (0.72-6.17)<br>1.78 (0.61-5.18)	 Ref<br>0.35 (0.03-3.87)<br>3.39 (0.23-50.40)	 Ref<br>0.21 (0.01-3.35)<br>11.53 (0.27-492.63)
**Intramuscular Adipose Tissue**<br>Low (tertile 1)<br>Middle (tertile 2)<br>High (tertile 3)	 ref<br>0.63 (0.21-1.86)<br>1.36 (0.55-3.39)	 Ref<br>0.10 (0.10-1.05)<br>1.61 (0.36-7.23)	 Ref<br>0.11 (0.01-1.26)<br>1.73 (0.36-8.26)
**Visceral Adipose Tissue**<br>Low (tertile 1)<br>Middle (tertile 2)<br>High (tertile 3)	 ref<br>1.18 (0.43-3.28)<br>1.50 (0.57-3.93)	 Ref<br>0.75 (0.13-4.48)<br>2.63 (0.30-22.84)	 Ref<br>0.83 (0.14-4.98)<br>1.39 (0.32-6.07)
**Very-Low-Density Muscle**<br>Low (tertile 1)<br>Middle (tertile 2)<br>High (tertile 3)	 ref<br>0.87 (0.35-2.18)<br>0.71 (0.25-2.03)	 Ref<br>1.94 (0.17-22.25)<br>1.52 (0.13-17.72)	 Ref<br>1.59 (0.13-19.71)<br>0.99 (0.06-17.27)
**Low-Density Muscle**<br>Low (tertile 1)<br>Middle (tertile 2)<br>High (tertile 3)	 ref<br>1.03 (0.37-2.88)<br>1.46 (0.54-3.97)	 Ref<br>5.36 (0.43-67.24)<br>6.21 (0.43-89.57)	 Ref<br>9.43 (0.60-149.12)<br>0.93 (0.35-147.42)
**Normal-Density Muscle**<br>Low (tertile 1)<br>Middle (tertile 2)<br>High (tertile 3)	 ref<br>0.46 (0.16-1.36)<br>0.86 (0.35-2.15)	 Ref<br>8.33 (0.58-119.93)<br>0.79 (0.28-51.90)	 Ref<br>6.98 (0.46-106.65)<br>5.26 (0.34-81.41)
**High-Density Muscle**<br>Low (tertile 1)<br>Middle (tertile 2)<br>High (tertile 3)	 ref<br>0.92 (0.35-2.44)<br>1.16 (0.44-3.04)	 Ref<br>2.01 (0.25-16.29)<br>1.86 (0.13-26.70)	 Ref<br>2.10 (0.24-18.46)<br>1.42 (0.08-25.13)
**Very-High-Density Muscle**			

Low (tertile 1)<br>Middle (tertile 2)<br>High (tertile 3)	ref<br>0.68 (0.24-1.93)<br>0.80 (0.32-2.01)	Ref<br>0.52 (0.05-5.21)<br>14.26 (0.65-311.83)	Ref<br>0.42 (0.03-5.00)<br>18.13 (0.49-669.03)
**Total Adipose Tissue**<br>Low (tertile 1)<br>Middle (tertile 2)<br>High (tertile 3)	 ref<br>2.21 (0.76-6.47)<br>1.95 (0.67-5.69)	 Ref<br>0.77 (0.07-8.28)<br>2.74 (0.20-36.73)	 Ref<br>0.38 (0.03-5.41)<br>3.46 (0.20-60.68)
**Total Skeletal Muscle**<br>Low (tertile 1)<br>Middle (tertile 2)<br>High (tertile 3)	 ref<br>0.73 (0.25-2.12)<br>2.08 (0.77-5.58)	 Ref<br>6.51 (0.45-95.19)<br>2.20 (0.21-23.11)	 Ref<br>13.36 (0.72-248.11)<br>2.48 (0.20-31.31)

[a] Model 2 is adjusted for age, race, disease stage, tumor grade, and tumor size

[b] Model 3 is adjusted for age, race, disease stage, tumor grade, tumor size, and adipose tissue (for skeletal muscles) or muscle (for adipose tissue)

TAT area = areas of SAT+IMAT+VAT

TSM area = areas of VLDM + LDM + NDM + HDM + VHDM

TNBC: triple-negative breast cancer

Table S2. Odds ratio and 95% CI for the association between specific body composition components and TNBC subtype among patients with post-surgery CT scans (N=229)

Body composition	Model 1 Unadjusted OR (95% CI)	Model 2 Adjusted OR[a] (95% CI)	Model 3 Adjusted OR[b] (95% CI)
**Subcutaneous Adipose**			
**Tissue**	Ref	Ref	Ref
Low (tertile 1	2.01 (0.93-4.36)	3.71 (1.09-12.64)*	6.00 (1.52-23.75)*
Middle (tertile 2)	3.13 (1.37-7.14)*	4.78 (1.22-18.71)*	11.15 (2.20-59.15)*
High (tertile 3)			
**Intramuscular Adipose**			
**Tissue**	Ref	Ref	Ref
Low (tertile 1)	1.59 (0.74-3.42)	2.31 (0.69-7.71)	2.67 (0.77-9.29)
Middle (tertile 2)	3.31 (1.42-7.72)*	8.79 (1.83-42.11)*	11.31 (2.11-60.76)*
High (tertile 3)			
**Visceral Adipose Tissue**			
Low (tertile 1)	Ref	Ref	Ref
Middle (tertile 2)	3.75 (1.68-8.37)*	4.36 (1.16-16.45)*	5.98 (1.43-24.92)*
High (tertile 3)	5.59 (2.39-13.10)*	24.69 (4.90-124.43)*	39.73 (6.45-244.87)*
**Very-Low-Density Muscle**			
Low (tertile 1)	Ref	Ref	Ref
Middle (tertile 2)	1.19 (0.52-2.69)	2.60 (0.68-9.88)	2.93 (0.75-11.47)
High (tertile 3)	2.01 (0.92-4.36)	4.25 (1.13-16.07)*	7.58 (1.54-37.25)*
**Low-Density Muscle**			
Low (tertile 1)	Ref	Ref	Ref
Middle (tertile 2)	2.37 (1.03-5.46)*	7.46 (1.83-30.34)*	4.31 (0.88-21.04)
High (tertile 3)	1.83 (0.86-3.92)	8.73 (2.11-36.09)*	3.86 (0.71-20.97)
**Normal-Density Muscle**			
Low (tertile 1)	Ref	Ref	Ref
Middle (tertile 2)	0.35 (0.15-0.82)	0.52 (0.14-1.95)	0.36 (0.08-1.54)
High (tertile 3)	0.31 (0.13-0.71)	0.17 (0.04-0.68)	0.16 (0.04-0.74)
**High-Density Muscle**			
Low (tertile 1)	Ref	Ref	Ref
Middle (tertile 2)	0.92 (0.38-2.21)	0.50 (0.12-2.15)	0.80 (0.14-4.69)
High (tertile 3)	0.94 (0.42-2.08)	0.25 (0.06-0.98)	0.45 (0.08-2.46)
**Very-High-Density Muscle**			
Low (tertile 1)	Ref	Ref	Ref

Middle (tertile 2)	1.02 (0.46-2.26)	0.72 (0.22-2.37)	0.74 (0.18-3.06)
High (tertile 3)	1.32 (0.60-2.91)	0.57 (0.18-1.83)	0.14 (0.03-0.72)
**Total Adipose Tissue**			
Low (tertile 1)	Ref	Ref	Ref
Middle (tertile 2)	1.50 (0.72-3.10)	2.93 (1.05-8.21)*	4.18 (1.30-13.46)*
High (tertile 3)	7.44 (2.62-21.14)*	22.19 (5.25-93.85)*	39.23 (7.67-200.58)*
**Total Skeletal Muscle**			
Low (tertile 1)	Ref	Ref	Ref
Middle (tertile 2)	2.00 (0.81-4.94)	0.89 (0.23-3.37)	0.49 (0.10-2.42)
High (tertile 3)	0.72 (0.34-1.51)	0.74 (0.24-2.29)	0.24 (0.06-0.98)

[a] Model 2 is adjusted for age, race, disease stage, tumor grade, and tumor size

[b] Model 3 is adjusted for age, race, disease stage, tumor grade, tumor size, and adipose tissue (for skeletal muscles) or muscle (for adipose tissue)

TAT area = areas of SAT+IMAT+VAT

TSM area = areas of VLDM + LDM + NDM + HDM + VHDM

TNBC: triple-negative breast cancer

*p <0.05

Table S3. Four-category of body composition phenotype in relation to TNBC subtype, by the timing of CT scan.

**Body composition categories**	**Model 1 Unadjusted OR (95% CI)**	**Model 2 Adjusted OR[a] (95% CI)**
**CT scan taken before surgery**		
(N=121)	Ref	Ref
Normal (Low adiposity, high muscle)	1.63 (0.28-9.41)	7.62 (0.28-208.09)
High adiposity, high muscle	1.67 (0.26-10.77)	2.91 (0.13-63.54)
Low adiposity, low muscle	1.00 (0.17-5.87)	3.13 (0.19-51.17)
High adiposity, low muscle		
**CT scan taken after surgery** ***(N=229)***		
Normal (Low adiposity, high muscle)	Ref	Ref
High adiposity, high muscle	0.27 (0.06-1.30)	0.26 (0.04-1.90)
Low adiposity, low muscle	0.36 (0.07-1.84)	0.56 (0.08-4.23)
High adiposity, low muscle	2.42 (0.36-16.36)	8.07 (0.74-88.21)

[a] Model 2 is adjusted for age, race, disease stage, tumor grade, and tumor size
